# A comparative study of the breathing pattern and amount of nasopharynx obstruction by the pharyngeal tonsil in hiv infected and non infected children

**DOI:** 10.1016/S1808-8694(15)30117-8

**Published:** 2015-10-19

**Authors:** Michella Dinah Zastrow, Liliane Janete Grando, Aroldo Prohmann de Carvalho, Inês Beatriz da Silva Rath, Maria Cristina Calvo

**Affiliations:** a M.S. Professor of Radiology and Stomatology, Unisul; b PhD. Adjunct Professor - Department of Pathology UFSC; c PhD. Adjunct Professor - Department of Pediatrics, UFSC; d PhD. Adjunct Professor - Department of Stomatology, UFSC; e PhD. Adjunct Professor - Department of Public Health, UFSC. Federal University of Santa Catarinha (UFSC)-Joana Gusmão Children’s Hospital - Florianópolis

**Keywords:** adenoids, child, hiv, radiography

## Abstract

Aim: the goal of the present investigation was to study the association between breathing pattern and pharyngeal tonsil size in 122 children (60 HIV infected and 62 without such infection). **Material and Methods:** The children were analyzed as to their breathing pattern, nasal flow and pharyngeal tonsil obstruction seen in side cephalometric x-rays, by means of a computerized analysis. **Results:** The pattern that most occurred in both groups was the mixed type. Most of the children presented oral or mixed type breathing and there was no association between the type of breathing and HIV presence (p=0.091). Nasal flow was mainly medium in both groups. Children without prior history of HIV infection had medium to large nasal flow and most of the HIV-infected children had medium nasal airflow. There was a positive association between nasal flow and HIV infection (p<0.0001). The average percentage of nasopharynx obstruction by the pharyngeal tonsil was high in both groups, and there was no statistically significance difference between them. Children from both groups had a moderate or large size of pharyngeal tonsil, and there was no association between tonsil size and HIV (p=0.21).

## INTRODUCTION

Tonsils, which are part of the Waldeyer’s lymphatic ring1, because of its strategic location, are the first host site that is exposed to inhaled or swallowed antigens, stimulating immune responses.^2^ Pharyngeal tonsils are multilobulated, and are located on the roof of the pharyngeal cavity^3^, which starts its development during the last months of fetal life, following a very fast growth pattern until three years of age. It reaches its largest volume at 7-10 years of age, and gradually reduces in volume with time and may even be totally absent in adults.^4^ It bears invaginations in the form of folds or clefts, covered by a non-uniform epithelium known as lympho-epithelium, which contains epithelial cells, lymphocytes, macrophages and dendritic cells.^5^

Pharyngeal tonsil (adenoids) enlargement may happen because of infectious-related hypertrophy (viral, bacterial or other); or hyperplasia (increase in cell count triggered by immunological needs).^2^, ^6^ Usually, tonsil and nasopharynx growth happens in harmony, and the increase in nasopharynx width is enough to accommodate the pharyngeal tonsil tissue that is growing, keeping the airway patent.^7^, ^8^, ^9^ However, if there is no balance, the result will be space reduction and nasopharyngeal obstruction, and oral breathing as a mechanism for survival.^7^, ^9^

Increase in pharyngeal tonsils may negatively impact developing children, resulting in a reduction in nasal air flow, upper airway obstruction, leading the patient towards oral or mixed complementary breathing patterns.^10^

When nasal breathing (physiological and vital) is partially or totally replaced, there is a change in the individual’s bodily organization, which can lead to morphological, functional, pathological, feeding and behavioral disorders.^6^ Oral breathing causes adenoid facies (narrow nose, prominent incisive teeth, high hard palate and open mouth), pulmonary and pharyngeal infections and chronic sinusitis.^11^

The most consequences to patients happen with an increase volume of the pharyngeal tonsil, and the therapeutic strategy is based not only on respiratory difficulties, but also because of the frequent concurring complications such as otitis, sinusitis and sleep apnea.^12^ It is believed that there is a cause and effect relation between oral breathing caused by an enlargement of the pharyngeal tonsil and cranial vertical development, and facial and dentition development as well.^13^ Most of the authors accept the theory that pharyngeal tonsil enlargement causes pharyngeal obstruction, thus oral breathing, leading the child to alter oro-facial muscles’ positions and that of the jaw. Such alterations influence mastication, swallowing and speech, and may very well cause skeletal alterations.^14^, ^15^

One of the most frequent clinical manifestations in HIV-infected children is recurrent upper airway infection. These recurrences are associated with an increase in pharyngeal tonsil volume enlargement, together with the establishment of complementary oral breathing, which may cause important alterations in the child’s life quality.^16^

AIDS is characterized by a severe dysfunction in the immune system of the HIV-infected individual. As it happens with other viruses, HIV infects the target cell, having a certain predilection for CD4+ cells, especially the T helper lymphocyte (LTh). Once infected by the virus, these cells start to malfunction until their complete destruction, when of the synthesis of new viral particles, from the use of its own enzymes. Immunodeficiency is brought about by the loss of LTh, which are responsible for the cellular immune system.^17^

There is a great deal of evidence suggesting that HIV-1-infected cells are sequestered in lymphoid tissues, such as the tonsils. Viral particles are collected and destroyed in germinative centers in their initial stages of infection. These observations indicate that the disease is active and progressive in lymphoid organs, while the infection is clinically latent for prolonged periods.^18^, ^19^

HIV-1-infected cells are found in the lympho-epithelium of pharyngeal tonsil invaginations. The lymphoid tissue in the nasopharynx contributes to the chronic replication of HIV-119, and the ThCD4+ lymphocytes and monocytes/macrophages play an important role in the interaction and spread of such infection. These cells, favorite HIV targets, serve as a reservoir and a virus spread vehicle.20 A child’s pharyngeal tonsil bears more lymphocytes than that of and adult. A study analyzed lymphocyte subgroups in secondary lymphoid organs, including the pharyngeal tonsil, in HIV-infected children and children without such infection history, there was a reduction in the total Th lymphocyte count and in CD4/CD8 ratio of the pharyngeal tonsil of HIV-infected patients. An increase in T-cytoxic and B-lymphocytes cell counts was also found, just as a 200% increase in the monocyte count in infected children. The pharyngeal tonsil tissue shows the immunodeficiency stage in HIV-infected patients, thus providing additional information regarding the development and response of infected patients.^21^ One study found an increase in the amount of pharyngeal tonsil tissue through MRI images in HIV-infected persons when compared to healthy counterparts, however, no relation between pharyngeal tonsil enlargement and the hematocrit, leucocytes and TCD4+ lymphocytes.^22^ Another study analyzed side view x-rays of the nasopharynx of 94 children infected by HIV and 34 without infection, identifying the adenoid-nasopharynx (AN) ratio and the relationship with the different stages of the disease. Statistically significant differences in the AN ratio were found among the groups, showing that the radiographic alterations in pharyngeal tonsil size in HIV-infected children are of great diagnostic value and is important in treatment planning.^18^

The high incidence of upper airway infections seen in HIV-infected children, especially recurrent chronic otitis media and suppurative otitis media, are included in the AIDS diagnostic criteria, which leads almost always to the clinical suspicion of upper airway obstruction.^16^

Taking these aspects into account, the present investigation aimed at studying the association between breathing pattern and nasopharynx obstruction by the pharyngeal tonsil in HIV-infected children, comparing the behavior of these two variables with those from a group of children without prior history of HIV infection.

## MATERIALS AND METHODS

### Study outline

Cross-sectional contemporary cohort^23^

### Sample selection

The sample was made up of a total of 122 children in the age range between 6 and 14 years of age, which was then divided in two groups. Group 1 was made up of (60) vertically HIV-infected children seen at the Department of Infectology of the Children Hospital of this city, from an universe of 500 children seen annually; and group 2 had 62 children without history of HIV infection who were freely taken to a service of dental radiography. The groups were very similar as to age and gender. Children without history of HIV-infection were of the same age (6 months more or less old) and gender of those in the study group, in an attempt to keep the greatest possible sample homogeneity, making it possible to have a control sample through the demand of children without a prior history of HIV-infection who sought the dental radiology ward. Age range selection criteria was based on the age range when the pharyngeal tonsil reaches its largest volume, ruling out children below six years of age, because of the difficulties in realizing the radiographic technique.7 Determining the number of children in the study group was carried out based on statistical calculations of sample, by using a 95% parameter and 5% confidence in a sampling error. We excluded the children with history of tonsillectomy who presented labial-palatine fissures. This investigation was carried out after being approved by the Ethics in Research Committee of the participating institutions. (Project # 255/04). Children from both groups participated in the research project by means of signing an informed consent by the children’s parents or guardians - after being properly advised about the procedures to be carried out.

### Interview, clinical history and clinical evaluation

In clinical history, the investigator questioned the parents and /or legal guardians as to patient behavior, mood, impatience, agitation, lack of concentration on activities, hyperactivity, aggressiveness, tiredness and day time sleepiness, because these symptoms may reflect alterations in the patient’s body caused by oral breathing. We also investigated respiratory issues, such as allergic rhinitis, sinusitis, bronchitis, constant colds, tonsillitis, and increase in pharyngeal tonsil size. As far as nocturnal habits are concerned, we investigated nighttime oral breathing, snoring, sleeping position, enuresis and drooling on the pillow. These data were collected based on Marchesan’s proposal.^10^

The investigator also assessed these children clinically, in order to evaluate the patient’s facial shape and the speech organs during rest. During the evaluation, with the patient comfortably seated having both feet on the floor, we observed some characteristics related to daytime oral breathing, those proposed by Marchesan, 10 such as the ones we discuss below:

a) lips:
  - whether they were closed, open or partially open;
  - if open, whether or not it was possible to seal (short upper lip, obtuse angle nose, short labial frenulum and diastema between the upper central incisive - Class II);
  - the lower lip if everted, if the upper lip is thin or if both are enlarged;
b) tongue: tongue protrusion during speech;
c) hard palate: if narrow or dome-like;
d) nose: was the base enlarged?, were the nostrils narrow or was there any septum deviation?;
e) open mouth breathing;
f) chin muscle tension, lower lip curved downwards.

### Respiratory pattern classification

Patients with histories of frequent snoring, nighttime drooling, sleeping on their stomachs and with open mouth were classified as mixed breathers. When besides these traits, the patient also presented three or more characteristics of daytime oral breathing - mentioned in the above item of clinical assessment, the patient was then classified as an oral breather. Patients without nocturnal habits and less than two day time breathing characteristics, he/she was classified as a nasal breather.

### Nasal flow assessment

For nasal flow assessment, we used the Altmann’s^24^ graded mirror, in order to check and see if air passed thorough the nose and check for patency of both nasal cavities, related to the quality of water vapor exhaled and condensed on the mirror’s surface, and check to see if air was symmetrically exhaled from both nostrils, or if it was greater from one of them. This mirror, a graded metal plate, comes with a reference notepad of the same shape and size, used to write the findings from each patient. Before breathing assessment, the patient blew his/her nose strongly, one nostril after the other. The mirror was than placed by the investigator right below the nose, centralized, at the height of the anterior nasal spine (ANS). The patient must put his/her head up straight and close the lips during the evaluation. The mirror is kept right under the nose and after two exhales we marked the fog region with a marker on the mirror. After outlining it, we transferred the data to the reference paper pad by placing it over the mirror and copying the fog shape directly, by transparency.

In the mirror and in each sheet of the reference notepad there were the same milimetric grading, divided in equal size squares. Each square had 10mm on each side. When the patient had up to 30mm of anterior nasal flow he/she was classified as low flow; from 30 to 60mm, medium flow; and from 60 to 90mm, large flow. Some patients had no flow at all. From this classification we also clustered the groups of normal nasal air flow (large flow) or altered (medium, low or no flow at all) flow in order to do the statistical analysis in comparison to the size of the pharyngeal tonsil and respiratory pattern.

### X-rays

The children underwent side view cephalometric radiographies, with a lead vest protection, by means of using the J. MORITA VERAVIEW ® (Kyoto, Japan) panoramic x-ray machine.

The x-ray was taken with the patient seating upright, with the head in a cephalostate with the supports on the external auditory canal and the Frankfurt Horizontal View (FHV) parallel to the soil. Exposures were carried out using 62 to 66kV and from 8 to 10mA. Exposure time varied between 0.8 and 1.0 seconds. These variations in exposure happened according to patient’s age, size and bone structure. The distance between the x-ray source and the child’s head was of one meter and fifty-two centimeters (1.52cm).

After exposure, the x-ray films were processed in a REVELL ® (São Paulo - SP, Brazil) automatic processing machine, in the dark chamber of the Radiology Department, using 2.5 minutes of total processing time.

### Interpretation - Calibration

One examiner - a radiologist, was trained to assess the radiographies obtained. This training was based on the evaluation of ten (10) radiographies to start with, under all the ideal interpretation conditions.^25^ After 7 (seven) days; the same ten radiographies previously analyzed were reassessed by the same examiner, with a 95% concordance and a 5% intra-examiner error.

The films from both groups were numbered and masked by another professional so as to keep him from identifying the patient and prevent an eventual biased analysis by the examiner.

### X-Ray evaluations

On the images obtained by the side-view cephalometric radiographies, previously masked and numbered, we reproduced the anatomical structures of interest in acetate paper, with help of a transmitted light viewer in a dimly lit room, with the help of masks, allowing for the later identification of reference points and to trace lines and planes that helped analyze the nasopharyngeal space.^25^

**Figure 1:**
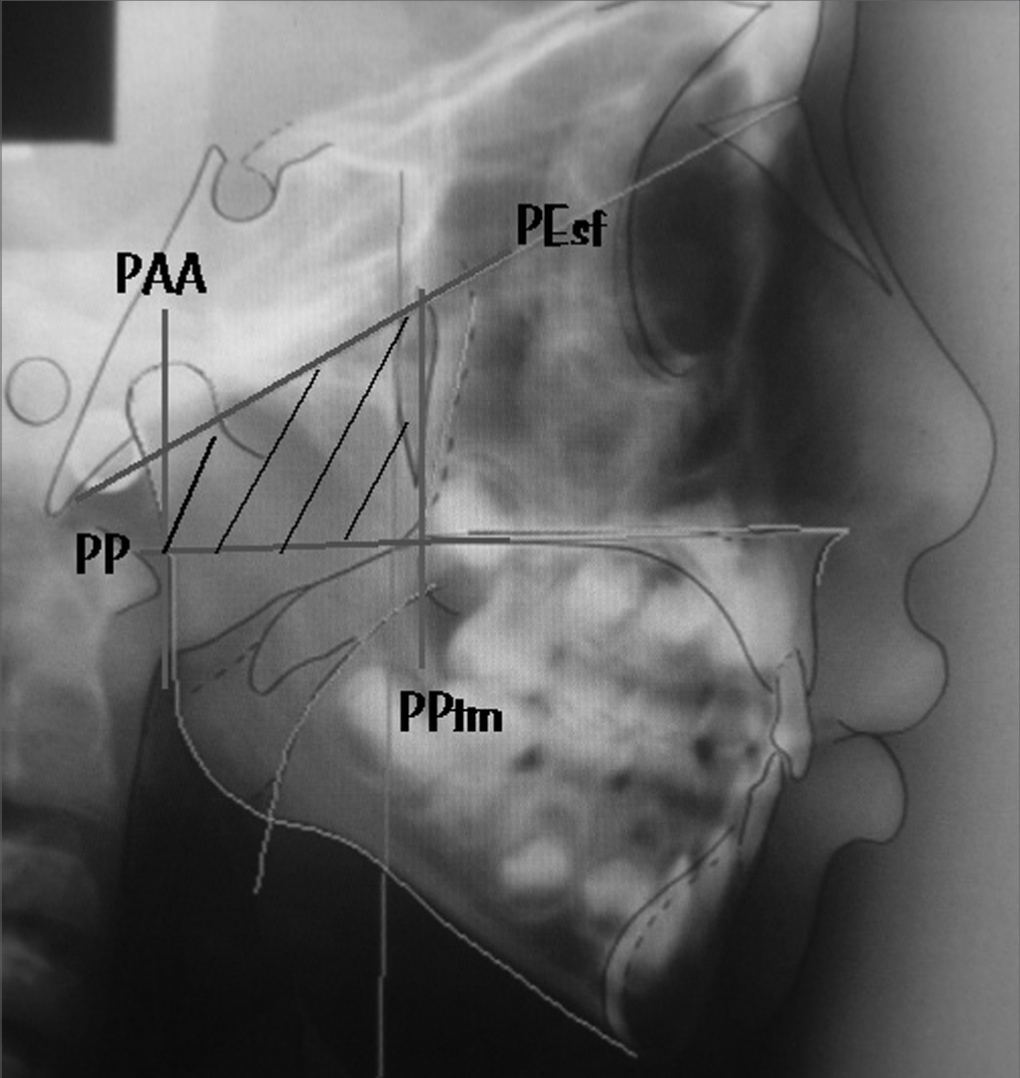
Planes that define the trapezoid used to calculate the nasopharynx area: palatal plane (PP), sphenoidal plane (Pesf), plane going through AA (PAA) and plane going through ENP (PPtm).

After marking on the film, we scanned the radiograph by using an HP Scanjet 4C/T ® scanner (São Paulo - SP, Brazil) and the image was exported to a computerized cephalometrics software. In order to identify the level of airway obstruction sideways and to determine the nasopharyngeal space occupied by the pharyngeal tonsil we used the adenoid percentage measure advocated by Handelman; Osborne (1976)^7^ and studied by Poole; Engel; Chaconas (1980)^26^ and Ricketts et al (1998)^27^. We used the trapezoid area calculation to obtain the total nasopharyngeal space. The four trapezoid sides are represented by four planes: the palate plane (PP), which goes through the anterior nasal spine (ANS) and the posterior nasal spine (PNS); the sphenoid plane (SphP), which goes through the basion point (Ba) and passes tangentially to the sphenoid bone; the plane that goes through the most anterior portion of the Atlas vertebrae (AA) [PAA] and finally, the pterigomaxillary plane (PPtm), a plane that is perpendicular to the palatal plane, passing by Ptm/ENP (Figure 1). The pharyngeal area is mathematically derived by using the nasopharyngeal depth (d), the nasopharyngeal height (h) and the angle formed between the sphenoid plane and the palatal plane (θ). The aerial portion is measured by means of a planimetric polar compensation; and the portion occupied by the pharyngeal tonsil is obtained by subtracting the aerial portion from the total nasopharyngeal area. For such analysis, we used the modified “Adenoid studies” from the Radiocef software, of Radiomemory® (Belo Horizonte - MG, Brazil), which calculates the percentage of nasopharyngeal obstruction caused by the pharyngeal tonsil.

A pharyngeal tonsil that occupied less than half of the nasopharyngeal area (≤ 50%) was considered normal; of moderate size would be those that occupied between 50 to 75% of the nasopharynx; and of marked size those that occupied more than 75% of the nasopharyngeal space.^28^

### Surveying the charts from group I children

By analyzing the charts of patients with HIV infection history, we established the values for TCD4+ and CV lymphocytes, they were all carried out in the same laboratory through the same assessment method. Most of these exams are periodically held every 3 to 4 months, in order to clinically control the patient and the assessment of the treatment administrated.

We also investigated the therapeutic scheme used by the children in the study.

### Statistical Evaluation

The results obtained were submitted to a descriptive statistical analysis; and the Mann-Whitney test was used to compare groups 1 and 2 as to nasopharynx obstruction by the pharyngeal tonsil, and the chi-squared to check for the possible associations between the virus presence and the type of breathing, nasal flow and nasopharyngeal obstruction by the pharyngeal tonsil.^23^

## RESULTS

Of the 122 children studied, 69 were females and 53 were males. Age ranged from 6 years and 1 month to 14 years. Age average in both groups was of 9 years and 8 months. Both groups were very similar as to age (p=0.945) and were not different as to gender (p=0.733).

The most frequent respiratory pattern in both groups was the mixed one, followed by nasal breathing and oral breathing (Table 1). Chi-squared test indicated that there was no association between breathing pattern and HIV presence (p=0.091).

**Table 1:** Number of children according to nasal airflow, Breathing pattern and presence (Group 1) or absence (Group 2) of HIV infection. Florianópolis, SC, 2006.

CRITERIA ASSESSED		GROUP 1		GROUP 2		TOTAL		x^2^
		n	%	n	%	n	%	
NASAL FLOW (a)	Absent	2	3,3	-	-	2	1,6	p<0,0001
	Little	18	30,0	-	-	18	14,8	
	Medium	31	51,7	42	67,7	73	59,8	
	Large	5	8,3	20	32,3	25	20,5	
BREATHING (b)	Oral	19	31,7	12	19,4	31	25,4	p=0,091
	Mixed	18	30,0	31	50,0	49	40,2	
	Nasal	19	31,7	19	30,6	38	31,1	

Total		60	100,0	62	100,0	122	100,0	

(a) and (b): see classification in materials and methods.

Nasal airflow was medium in both groups, according to Table 1. The chi-squared test indicated an association between airflow and HIV presence (p<0.0001). There was less airflow in the HIV-infected children. Four children (4) were taken off group 1 (6.7%) because they did not come for nasal flow assessment.

The average percentage obstruction caused by the pharyngeal tonsil in group 1 was of 70.37% ± 14.07 and in group 2 it was of 67.80% ± 10.24. Percentage was high in both groups, and there was no significant difference between them. Moderate obstruction prevailed in both groups (Table 2). The chi-squared test indicated that there was no association between pharyngeal tonsil size and HIV (p=0.201). The Mann-Whitney test also did not indicate statistically significant difference between the two groups (p=0.09).

**Table 2:** Number of children according to nasopharynx obstruction percentage by the pharyngeal tonsil (PT) and presence (Group 1) or absence (Group 2) of HIV infection. Florianópolis, SC, 2006.

% OF OBSTRUCTION CAUSED BY THE PHARYNGEAL TONSIL	GROUP 1		GROUP 2		TOTAL		TEST
	n	%	n	%	n	%	
Normal	5	8,3	2	3,2	7	5,7	p=0,201
Moderate	34	56,7	44	71,0	78	63,9	
High	21	35,0	16	25,8	37	30,3	

AVERAGE (SD)	70,37%	(14,07)	67,80%	(10,24)			p=0,09

Since we group and classify the breathing pattern and nasal air flow in two categories only, results showed that in both groups, children distribution was greater in the oral or mixed breathing pattern and nasal air flow (absent, little or medium) (Table 1). There was also no association between tonsil obstruction percentage, breathing and air flow alterations (Table 3).

**Table 3:** Number of children according to nasopharynx obstruction by the pharyngeal tonsil (PT) and air flow breathing alterations, in the children with HIV infection (Group 1) or without (Group 2). Florianópolis, SC, 2006.

HIV	AIR FLOW AND/OR BREATHING PATTERN	% OF OBSTRUCTION BY THE PHARYNGEAL TONSIL				x^2^
		Intense	Moderate	Normal	Total	
GROUP 1	With alterations	20	32	5	57	p=0,8516
	Without alterations	1	2	-	3	
	Total	21	34	5	60	
GROUP 2	With alterations	12	38	2	52	p=0,4683
	Without alterations	4	6	-	10	
	Total	16	44	2	62	
TOTAL	With alterations	32	70	7	109	p=0,5584
	Without alterations	5	8	-	13	
	Total	37	78	7	122	

In Group 1, the average TCD4+ lymphocytes percentage was of 35.01% ± 10.76 and the CV mean logarithm value was of 3.25 ± 1.08. The values considered ideal for a clinical presentation without symptoms included a relative TCD4+ lymphocyte count above 25% and the CV logarithm (log CV) below 2.00. Of the sixty HIV-infected children, 58 (96.7%) were under some type of retroviral treatment. Only two (3.3%) were not submitted to any type of treatment since they were clinically and immunologically stable.

## DISCUSSION

Almost 90% of all the children, including those children with and without an HIV-infected history had some kind of breathing alteration, without reduction on nasal airflow, breathing pattern alteration (oral or mixed), or both. Of these children, less than 1% had a normal percentage of pharyngeal tonsil obstruction. Nonetheless, all the children who did not have such alterations had their pharyngeal tonsils obstructing more than 50% of the space. These results show that in the children investigated the alterations seen are not in agreement with most of the papers in the literature.^6^, ^11^, ^12^, ^15^, ^29^, ^30^, ^31^, ^32^

There are numerous methods which are considered specific for the assessment of nasal permeability, among them we have the expiratory flow metering mirror (including the milimetrically graded Altmanns mirror), rhinomanometry and acoustic rhinometry.^33^ The most accurate and recent included are: acoustic rhinometry and rhinomanometry. All respiratory tests have limitations, since they do not produce enough information that can state for certain whether the patient is an oral breather, therefore, it is necessary to complete the data obtained through the anamnesis and the clinical exam.^34^ Rhinomanometry is not broadly available, especially when we consider the economic reality of the service where the study was carried out, and since it is an invasive type of exam, it would require an otorhinolaryngologist present during the exam, and it was not possible to have one. Having all these facts in mind, we chose to use Altman’s graded nasal mirror, a test that is no as accurate as rhinomanometry, however simple and of low cost, available in our service, easily accepted by the children and that allowed a study of the sample.

Many signs and symptoms associated with oral or mixed breathing were found among the children studied. The most common were snoring, nocturnal drooling, upper lips underdeveloped, high hard palate, narrow nostrils and tension in chin muscles, already mentioned by Nishimura (2003)^35^ and Kobayashi, (2003).^36^

HIV-infected children had more nasal air flow alterations (91.1% of the children in the sample), showing that the flow alteration is not related only to the size of the pharyngeal tonsil. These children have a higher frequency of repetition upper airway infections (UAI), especially repetitive colds, which may also cause nasal obstructions, even if temporary only, reflecting in nasal air flow alteration.^31^

The results of the present investigation do not show statistically significant differences in the amount of nasopharynx obstruction by the pharyngeal tonsil in the two groups studied. These results are not in agreement with the results from Yousem et al. (1997)^22^, when differences were found in tonsil size in patients with and without prior history of HIV-infection. Yousem et al’s. (1997)^22^ studied adult patients with mean age of 37 years, not being restricted to children only. As it is known, the pharyngeal tonsil gradually reduces in size after puberty (around 10 to 12 years of age).^4^ Therefore, most healthy adult patients have total reduction of their pharyngeal tonsil; and this lack of agreement between the present study’s result with the study by Yousem et al. (1997)^22^ may represent a reflex of this difference in samples. Moreover, Yousem et al. (1997)^22^ used MRI images, which is considered much better than side view cephalometric radiographies in the assessment of soft tissue.

In the present investigation we tried to obtain samples that were similar as far as age and gender are concerned, in an attempt to obtain a homogeneous sample and control age-related alterations, which is fundamental in the assessment of pharyngeal tonsil. As far as gender is concerned, hormonal alterations between men and women are also some of the factors that may influence tonsil size.^8^

Benito et al. (1999)^18^ also reported statistically significant differences as to pharyngeal tonsil size in the groups studied - HIV-infected and non-infected children. Differences in results may reflect a possible relation between children’s age at the aforementioned study, which are very young when compared to the present study. The age range of these children varied between 6 months and 15 years, with more children below 7 years of age, age in which the pharyngeal tonsil still has not reached its growth peak. As to the disease’s clinical stage and immunodeficiency, we noticed that the children of the present study have their immune system under strict control with the use of antiretroviral medication (proven by control blood exams). There was no difference in terms of pharyngeal tonsil size in the groups studied, since almost all children from Group 1 with HIV, except for two (3.3%) use the medication, with good treatment compliance; and the pharyngeal tonsil enlargement may be considered physiological for that age. Therefore, pharyngeal tonsil enlargement may be considered a physiological immune response to HIV-infection stimulus, as it happens with other diseases.

Proper treatment compliance greatly influences treatment results, both in adults and in children infected by the HIV, bringing about viral replication control and immune system recovery. As for proper treatment compliance we understand that patients take the antiretroviral medication correctly, follow the correct doses for the pre-established amount of time, and comply with the health care service provided. Children compliance goes through the hands of the caretaker, who has to be educated about the importance of his/her participation in the treatment and be well instructed about possible treatment difficulties. This is one of the determining factors in order to guarantee a good response to HIV-infection treatment.^37^

The methodology employed by the present investigation is based on the assessment of nasopharynx obstruction by calculating the area, which seems to be more accurate and encompassing than a linear evaluation as the AN measure, used in the study by Benito et al. (1999).^18^ Differences in these two methods of assessment may impact the final results of the studies, and it may show statistically significant differences between the studies. By using a linear measure, many things may happen: we may be facing a large pharyngeal tonsil and the nasopharyngeal space be large enough to accommodate it and, therefore, it may not negatively impact the patient’s breathing; as well as a small tonsil in a large space; or, one may have a small tonsil, in a small nasopharynx, causing obstruction, however a large enough tonsil may also cause obstruction. Studies comparing the two assessment methods are necessary in order to find data that show whether there are statistically significant differences between them.

## CONCLUSIONS

The results achieved let us draw the following conclusions:

1. Children with and without HIV-infection history, with ages between 6 and 14 years, had moderate to high percentage of nasopharynx obstruction by the pharyngeal tonsil, and there was no association between pharyngeal tonsil enlargement and HIV infection.
2. Most children had oral or mixed breathing pattern, and there was no association between breathing pattern and HIV infection.
3. Children without history of HIV infection had altered (middle) nasal flow and most children with HIV-infection history also had altered nasal air flow (little to medium), and there was an association between nasal airflow and HIV.
